# The Exploring Functional Role of Ammonium Transporters of *Aspergillus oryzae* in Nitrogen Metabolism: Challenges towards Cell Biomass Production

**DOI:** 10.3390/ijms23147567

**Published:** 2022-07-08

**Authors:** Chanikul Chutrakul, Sarocha Panchanawaporn, Tayvich Vorapreeda, Sukanya Jeennor, Jutamas Anantayanon, Kobkul Laoteng

**Affiliations:** 1 Functional Ingredients and Food Innovation Research Group (IFIG), National Center for Genetic Engineering and Biotechnology (BIOTEC), National Science and Technology Development Agency (NSTDA), Thailand Science Park, Phahonyothin Road, Khlong Nueng, Khlong Luang, Pathum Thani 12120, Thailand; sarocha.pan@biotec.or.th (S.P.); sukanya.jee@biotec.or.th (S.J.); jutamas.ana@biotec.or.th (J.A.); kobkul@biotec.or.th (K.L.); 2 Biochemical Engineering and Systems Biology Research Group (IBEG), National Center for Genetic Engineering and Biotechnology (BIOTEC), National Science and Technology Development Agency (NSTDA), at King Mongkut’s University of Technology Thonburi, Bangkok 10150, Thailand; tayvich.vor@biotec.or.th

**Keywords:** *Aspergillus oryzae*, ammonium transporter, nitrogen metabolism, filamentous growth, overexpression

## Abstract

Ammonium is a source of fermentable inorganic nitrogen essential for the growth and development of filamentous fungi. It is involved in several cellular metabolic pathways underlying nitrogen transport and assimilation. Ammonium can be transferred into the cell by an ammonium transporter. This study explored the role of ammonium transporters in nitrogen metabolism and cell biomass production in *Aspergillus oryzae* strain BCC 7051. Specific sequences encoding ammonium transporters (Amts) in *A. oryzae* were identified using genomic analysis. Four of the identified ammonium transporter genes, *aoamt1*-*aoamt4*, showed similarity in deduced amino acid sequences to the proteins in the ammonium transporter/methylammonium permease (AMT/MEP) family. Transcriptional analysis showed that the expression of *aoamt2* and *aoamt3* was ammonium-dependent, and was highly upregulated under ammonium-limited conditions. Their functional roles are characterized by genetic perturbations. The gene disruption and overexpression of *aoamt3* indicated that the protein encoded by it was a crucial ammonium transporter associated with nitrogen metabolism and was required for filamentous growth. Compared with the wild type, the *aoamt3*-overexpressing strain showed superior growth performance, high biomass yield, and low glucose consumption. These results shed light on further improvements in the production of potent bioproducts by *A. oryzae* by manipulating the ammonium uptake capacity and nitrogen metabolism.

## 1. Introduction

Nitrogen is a crucial nutrient element for all living organisms, involved in the biosynthesis of basic biomolecules, including proteins, nucleic acids, and carbohydrates, and plays a role in the secondary metabolism of several fungi [1]. Nitrogen metabolism involves the incorporation of inorganic nitrogen into organic compounds to further synthesize the above-mentioned molecules. The metabolic pathways involved include nitrogen transport and assimilation, resulting in glutamic acid and glutamine production [2]. Glutamic acid plays a central role in the nitrogen metabolism in cells. Generally, microorganisms have a mechanism called nitrogen metabolite repression (NMR) to choose the best nitrogen source(s) and prevent the use of non-preferred nitrogen. In filamentous fungi, NMR is manipulated during growth on preferred nitrogen sources by the global nitrogen transcription factor (AreA) [2]. It is considered the major activator involved in utilizing alternative nitrogen sources when the preferred ones are absent [3]. This activator is responsible for regulating nitrogen uptake and the assimilation system relevant to nitrogen metabolism [4,5,6,7].

Although filamentous fungi can uptake both ammonium (NH_4_^+^) and nitrate (NO_3_^−^), ammonium is the preferred inorganic nitrogen source for cell growth [8,9]. Indeed, they render a variety of adaptation mechanisms in response to available or limited ammonium in surrounding regimes. Ammonium can be directly taken up by fungal cells through ammonium transporters (Amts). Alternatively, it can be derived from the reduction of nitrate by the cell, which is then assimilated into glutamic acid and glutamine biosynthesis via the glutamine/glutamate synthase (GS/GOGAT) cycle by glutamate dehydrogenase (GdhA), glutamine synthetase (GlnA), and glutamate synthase (GltA) [10]. These synthesized amino acids are precursors or donors of other organic molecules involved in cell growth, defense mechanisms, and pathogenicity [1,11]. To date, studies have emphasized the mechanisms of ammonium transport and regulation in several yeasts and fungi [2,5,7,9].

Ammonium transporters that mediate the uptake of ammonium into cells are structural membrane proteins that are members of the multigenic ammonium transporter/methylammonium permease (AMT/MEP) family [12,13,14]. These membrane proteins share consensus amino acid sequences among bacteria, fungi, plants, and animals, indicating conservation of this transporter family across living species. Over 1200 members of the AMT/MEP family obtained from more than 350 species of the kingdom of fungi have been discovered based on amino acid sequence similarity (Pfam accession number PF00909 (http://pfam.xfam.org/family/pf00909#tabview=tab0, accessed on 4 February 2022). According to functional characterization of ammonium transporters, the AMT/MEP/family in filamentous fungi contains three to four members [15,16,17,18]. A well-known study described AMTs in *Aspergillus nidulans*, which includes four transporter proteins, MepA, MepB, MepC, and MeaA, encoded by the *mepA*, *mepB*, *mepC*, and *meaA* genes, respectively [15,16,19].

*Aspergillus oryzae* is categorized as a food-grade cell factory and has been listed as Generally Recognized as Safe (GRAS). It widely used to produce traditional Asian foods and animal feed additives. The growth ability of *A. oryzae* in diverse carbon and nitrogen substrates and environmental conditions (i.e., pH and temperature) is advantageous for the production of valuable products with applications in the food, feed, and pharmaceutical industries, such as hydrolytic enzymes, functional proteins, lipids, organic acids, and secondary metabolites [20,21,22]. Some metabolites are growth-associated, and their productivity is usually affected by growth rate and biomass titer. With the advent of modern biotechnology, strain improvement is an approach of interest for enhancing nitrogen uptake directed at either cell biomass or targeted metabolite production. Nevertheless, better understanding of nitrogen transporters and metabolism in *A. oryzae* is required for industrial purposes and fundamental research on fungal physiology. In this study, we aimed to characterize the genes encoding ammonium transporters in the *A. oryzae* strain BCC 7051 (*aoamts*) and investigate their functional roles in ammonium uptake and assimilation processes relevant to growth phenotypes. Putative *aoamt* sequences of *A. oryzae* were identified by genome analysis, and their transcriptional profiles were analyzed by reverse-transcription real-time quantitative PCR (RT-qPCR). Functional characterization of the targeted *aoamts* was implemented by genetic perturbation. In addition, overexpression of a selected *aoamt* gene was performed to identify an attributive transporter for ammonium involved in the cell growth and enhanced biomass production. This study demonstrates the potential exploitation of the ammonium transport machinery of *A. oryzae* for further development of the fungal strain through synthetic biology approach for industrial application.

## 2. Results and Discussion

### 2.1. Identification and Characterization of the Ammonium Transporter Sequences of A. oryzae

**Figure 1:**
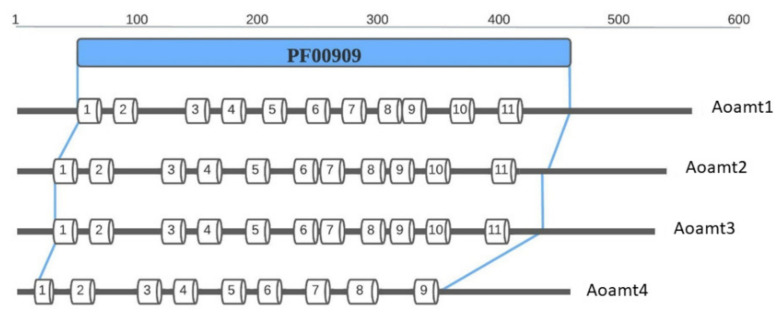
Transmembrane domains of Aoamt1, Aoamt2, Aoamt3, and Aoamt4 of *A. oryzae* BCC 7051. The positions of putative transmembrane (TM) helices and topology were assigned by using the TMHMM2.0 program [24].

Putative ammonium transporter genes in *A. oryzae* BCC 7051 were identified using genomic data [23]. Four *aoamt* genes were designated *aoamt1*, *aoamt2*, *aoamt3*, and *aoamt4*, with sequence lengths between 1.39 and 1.69 kb. The cDNA sequences were cloned and deposited in GenBank (http://www.ncbi.nlm.nih.gov, accessed on 30 March 2022). The deduced amino acid sequences of these *aoamt* genes were designated as Aoamt1 (OOO04540.1), Aoamt2 (OOO09170.1), Aoamt3 (OOO 606.1), and Aoamt4 (OOO14013.1). Based on amino acid sequence similarity analysis, the identified proteins were categorized as AMT/MEP family members containing the ammonium transporter family domain (PF00909). The amino acid sequences of Aoamt1 and Aoamt2 shared 89% (93% similarity) and 87% identity (92% similarity) with the MeaA and MepA proteins of *A. nidulans*, respectively. The amino acid sequences of Aoamt3 and Aoamt4 displayed 70% (85% similarity) and 86% identity (91% similarity) with MepB and MepC of *A. nidulans*, respectively. Among these, Aoamt1, Aoamt2, and Aoamt3 contain 11 transmembrane helices with an N_(out)_—C_(in)_ topology (Figure 1) based on the TMHMM program [24] in agreement with the conserved characteristic of other fungal ammonium transporters [18,25,26]. However, Aoamt4 is a distinctive AMT/MEP that contains nine transmembrane helices with an N_(out)_—C_(in)_ topology.

**Figure 2:**
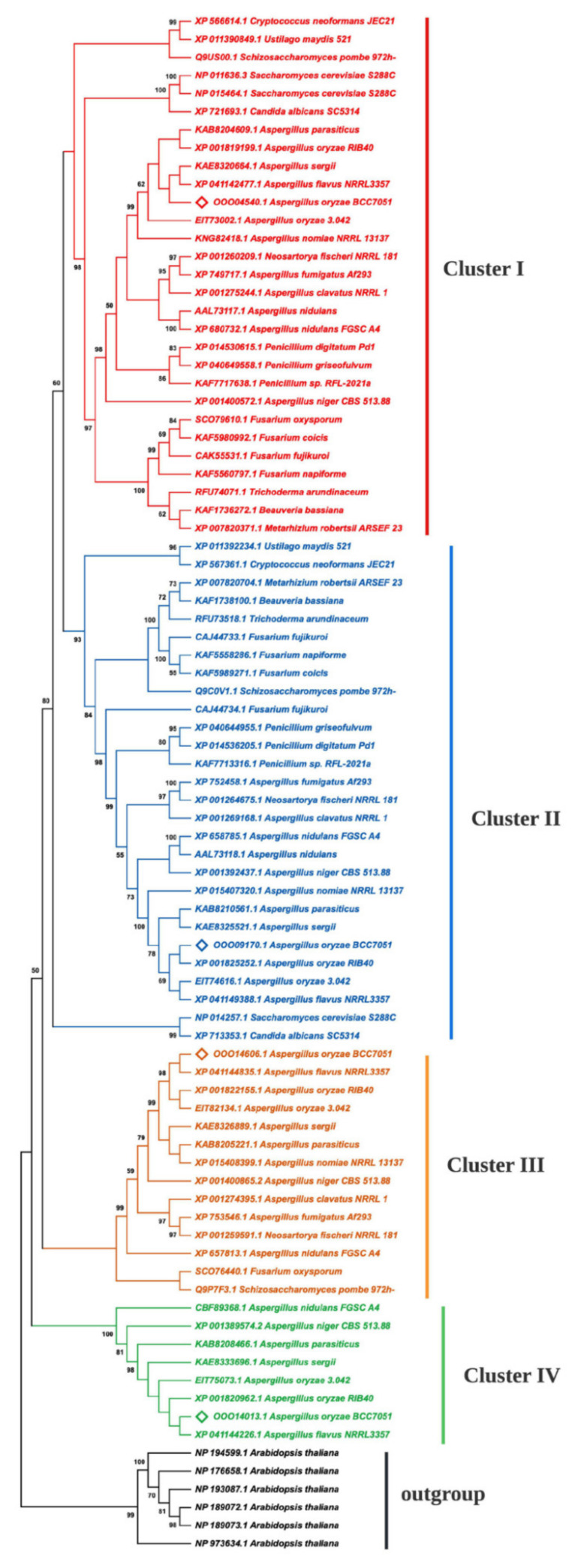
Phylogenetic dendrogram of the ammonium transporters of *A. oryzae* BCC 7051, yeasts, and fungi. The number over the nodes represents the bootstrap coefficient calculated from 500 replicates. Four AMT/MEP clusters, Cluster I, Cluster II, Cluster III, and Cluster IV, are highlighted in red, blue, orange, and green, respectively. (◊) indicates ammonium transporter sequence obtained from *A. oryzae* BCC 7051. The phylogenetic tree was constructed by the maximum-likelihood method using MEGA11 software.

Based on the maximum-likelihood method, a phylogenetic tree of Aoamt1, Aoamt2, Aoamt3, and Aoamt4 of *A. oryzae* and other members of the AMT/MEP family of yeasts and fungi, including 79 sequences from 28 species, is shown in Figure 2. The consensus tree, measured by bootstrap analysis of 500 random sequence replicates, revealed that the yeast and fungal AMT/MEP sequences were grouped into four clusters (Clusters I–IV). The amino acid sequences of *A. oryzae* BCC 7051 were clustered based on the phylogenic results and were distributed into four clusters. Aoamt1, Aoamt2, Aoamt3, and Aoamt4 are presumed to be MeaA-like, MepA-like, MepB-like, and MepC-like proteins, respectively. Aoamt1 belonging to Cluster I was highly similar to MeaA (XP_680732.1), Ump1 (XP_011390849.1), and Mep1 (NP_011636) and Mep3 (NP_015464.1) of *A. nidulans* [15], *Ustilago maydis* [27], and *Saccharomyces cerevisiae* [28], respectively. The Aoamt2 sequence was similar to that of the protein members in Cluster II, which were discovered in *A. nidulans* (MepA; XP_658785.1) [15], *U. maydis* (Ump2; XP_011392234.1) [27], and two species of yeast-like fungi, *S. cerevisiae* (Mep2; NP_014257.1) [28] and *Candida albicans* (Mep2; XP_713353) [29]. Aoamt3 was categorized in Cluster III. The protein members of this cluster were identified in 13 fungal strains and one yeast strain. Interestingly, Aoamt4, which was categorized in Cluster IV, was found particularly in *Aspergillus* spp. Members of this cluster rendered a unique characteristic, in which the length of amino acid sequences was shorter than that of other clusters resulting in nine transmembrane helices, as illustrated in Figure 1. Alignment of the deduced amino acid sequences of the four putative ammonium transporters of *A. oryzae* BCC 7051 indicated that Aoamt4 shared only 34–40% identity and 52–58% similarity with Aoamt1, Aoamt2, and Aoamt3. This result suggests that Cluster IV is more divergent than Clusters I, II, and III, which may have resulted from an early duplication independently lost in several fungal lineages as previously described [19]. Our data showed that AMT/MEP proteins in each cluster of *Aspergillus* spp. were varied in length and composition of amino acid residues in the C-terminal region (CTR). The CTR has been proposed as a regulatory region for ammonium transporters. So far, the structure-function relationship of this region has been reported. The crystal structure of plant (*Arabidopsis thaliana*) and prokaryotic (*Archaeoglobus fulgidus*) ammonium transporters suggests that the C-terminus interacts physically with the cytosolic loops of neighboring subunits in a phosphorylation-dependent manner [30,31]. In addition, the CTR mediates allosteric regulation of ammonium transport activity via phosphorylation [32,33,34]. Therefore, the variable length and amino acid composition of this region may affect the mechanism of action or transform the transporter into an ion channel and regulate the protein mediated by the CTR.

**Figure 3:**
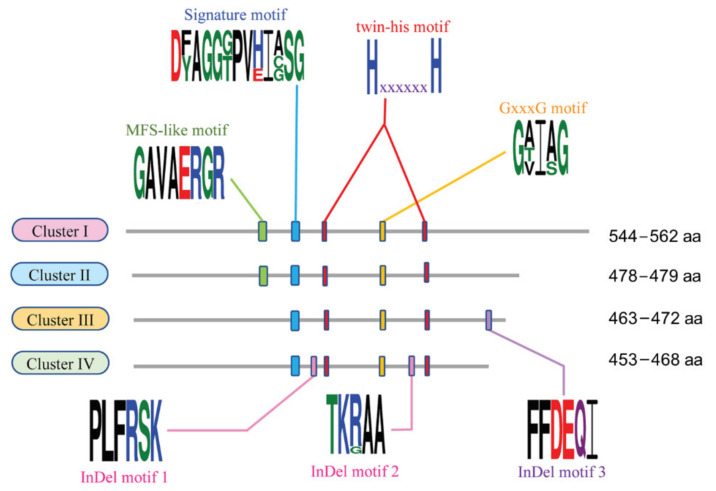
Distribution of consensus motifs in four clusters of *Aspergillus* ammonium transporters. The sequence logo was generated by WebLogo 3 (http://weblogo.threeplusone.com/, accessed on 11 March 2022) [41], showing signature features, including twin-his, GxxxG, MFS-like, and InDel motifs as indicated by blue, red, yellow, green, and pink boxes, respectively. The height of each letter is proportional to its frequency of occurrence. Hydrophobic residues are indicated by black letters. Acidic and basic amino acids are represented by red and blue letters, respectively. Polar and neutral residues are depicted by green and purple letters, respectively. “x” in the twin-his motif is defined as a non-specific amino acid residue. The number of amino acids (aa) in each cluster is shown.

The conserved Aoamts motifs of *A. oryzae* BCC 7051 were identified by multiple sequence alignment of *Aspergillus* AMT/MEP proteins. Three consensus motifs, the signature motif (DxAGGxPVxIxSG), twin histidine (twin-his), and GxxxG motifs, were present in all clusters, as shown in Figure 3. It has been postulated that the signature motif might be a region conferring a particular feature in the *Aspergillus* AMT/MEP family. The twin-his motif contained two conserved histidine residues with six non-specific amino acid residue (x) intervals. This core structure has also been proposed as a regulatory region responsible for the function and substrate conductance by mediating substrate deprotonation in AMT/MEP pores [35,36,37]. Together, the twin-his motif may have a significant impact on the kinetics of the AMT/MEP family transporters and a deleterious effect on selectivity, resulting in a switch from a specific transporter (NH4^+^ or NH3/H^+^) to an unspecific ion channel (K^+^) [26,35,38]. However, the signaling mechanisms involved remain unclear.

The GxxxG conserved motif has been proposed to be another regulatory region involved in protein folding that is essential for membrane channel formation [39,40]. Accordingly, this motif may play a critical role in regulating transporter pore size and is vital for generating the correct architecture for substrate binding and transport.

As shown in Figure 3, a major facilitator superfamily-like (MFS-like) motif with highly conserved amino acid residues of “GAVAERGR” (green boxes) was present only in Clusters I and II of the *Aspergillus* AMT/MEP family. This motif has been proposed to play a role in the translocation of ammonium across the membrane [16]. Amino acid substitution of the MFS-like motif found in Clusters III and IV presumably indicates an alternative conformation of the ammonium transporter to regulate its activity. Based on these results, we hypothesized that the MFS-like motif may refer to the evolutionary divergence of the *Aspergillus* AMT/MEP family. Multiple sequence alignment of the *Aspergillus* AMT/MEP members revealed short insertion/deletion residues (InDel motifs), in which InDel motifs 1 and 3 are present in Cluster III, while Indel motif 2 is found in Cluster IV (Figure 3, pink boxes). These conserved InDel motifs are observed only in the AMT/MEP members of *Aspergilli*, which may be involved in the function of the ammonium transporter.

### 2.2. Differential Expression of Ammonium Transporter Genes (Aoamts) in A. oryzae by Ammonium Treatment

**Figure 4:**
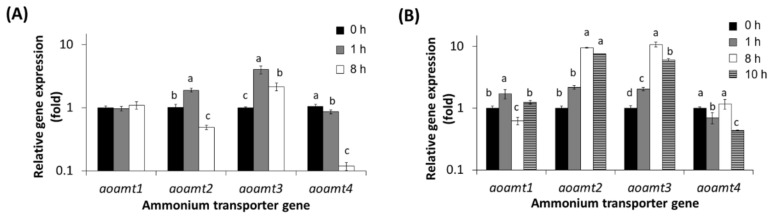
Differential expression of *aoamt* genes in *A. oryzae*. Expression analysis of *aoamt1*, *aoamt2*, *aoamt3*, and *aoamt4* genes of the wild-type cultures grown in modified Czapek Dox (mCD) medium in the presence of 1 mM NH_4_Cl (**A**) and 20 mM NH_4_Cl (**B**) was carried out by RT-qPCR. Total RNA was extracted from the cultures after medium transfer for 0, 1, 8, and 10 h (black, gray, white, and horizontal bars, respectively). The expression level of each gene at 0 h (black bars) was adjusted to 1. Different letters (a, b, c, and d) above the bars indicate a statistically significant difference in the transcript levels of each gene at various time points analyzed by Duncan’s multiple range test (MRT) (*p*-value < 0.05). The mean ± standard deviation (mean ± SD) of the expression level is derived from triplicate analysis.

Elucidating the differentially expressed genes (DEGs) involved in ammonium transport provides insights into how *A. oryzae* cells respond to the presence of ammonium. The transcriptional response of *aoamt* genes to ammonium-containing conditions was investigated in relation to ammonium uptake by fungal cells. After transferring the logarithmic culture of *A. oryzae* grown in modified Czapek Dox (mCD) medium to nitrogen-lacking medium followed by medium containing ammonium at different concentrations (1 mM and 20 mM NH_4_Cl), the residual ammonium concentrations in the culture broth at different cultivation times were quantified. The ammonium concentration declined to an undetectable level after transferring the culture to a 1 mM NH_4_Cl-containing medium for 1 h (Figure S1). The ammonium deficient stage was prolonged to 8 h on using 20 mM NH_4_Cl. Differential activation of *aoamt* genes during cultivation in the presence of 1 mM and 20 mM ammonium was observed and the transfer point of the cultures was used as a control (0 h). As shown in Figure 4, the transcript levels of the four *aoamt* genes were significantly affected by ammonium. Gene expression profiles can be divided into two groups: ammonium-sensitive and non-sensitive genes. Transcript analysis showed that *aoamt2*, *aoamt3*, and *aoamt4* expressions were sensitive to ammonium exposure. The expression level of *aoamt2* was detectable in the presence of ammonium and was significantly upregulated when ammonium was deficient, in contrast to the ammonium starvation condition. This was clearly observed after transferring the culture to a 1 mM NH_4_Cl-containing medium for 8 h (Figure 4A). The transcript level of *aoamt3* was also upregulated, similar to the *aoamt2* expression profile. However, the relative expression of *aoamt3* was maintained at an equivalent level during starvation, which was clearly observed in both media containing different NH_4_Cl concentrations (Figure 4A,B). These results do not coincide with the previous findings for *A. nidulans*, in which the *mepA* (*aoamt2*-like) expression continually increased when encountering ammonium starvation, and the expression of *mepB* (*aoamt3*-like) was detected only under complete nitrogen starvation conditions [19]. As shown in Figure 4, the ammonium starvation periods of the cultures using 1 mM and 20 mM NH_4_Cl were different after cultivation for 1 and 8 h, respectively. At the defined starvation stage, the different transcription profiles of *aoamt2* and *aoamt3* were found in both cultures. These results indicate that gene expression is not only dependent on the starvation stage but is also indirectly influenced by cultivation time, during which metabolic alteration might occur along with growth development, especially on prolonged cultivation (ammonium and other nutrient starvation). Moreover, it was found that the expression of *aoamt4* decreased during ammonium starvation. However, this result did not coincide with the *mepC* (*aoamt4*-like) of *A. nidulans*, whose expression increased under ammonium starvation conditions [19]. Our results revealed that the Aoamt4 protein encoded by *aoamt4* is an ammonium transporter required for cell growth and metabolism that is specific to ammonium availability.

It was found that the transcript levels of *aoamt1* in the cultures grown under ammonium-available and ammonium-limited conditions were not significantly different. However, a slight increase in *aoamt1* expression was observed under nitrogen starvation conditions. It is likely that this gene does not participate directly in ammonium uptake. This result was similar to that previously described for *A. nidulans*, in which *meaA* (*aoamt1*-like) was constitutively expressed in the presence and absence of ammonium [19]. Our study revealed the differential expression responses of *aoamt* genes in *A. oryzae* to ammonium-sufficient, ammonium-deficient, and ammonium-starvation conditions. The two ammonium-induced genes in *A. oryzae*, *aoamt2*, and *aoamt3* were selected for further study.

### 2.3. Assessing the Role of Ammonium Transporters in Phenotypic Growth on Solid Medium and Nitrogen Metabolism of A. oryzae

**Figure 5:**
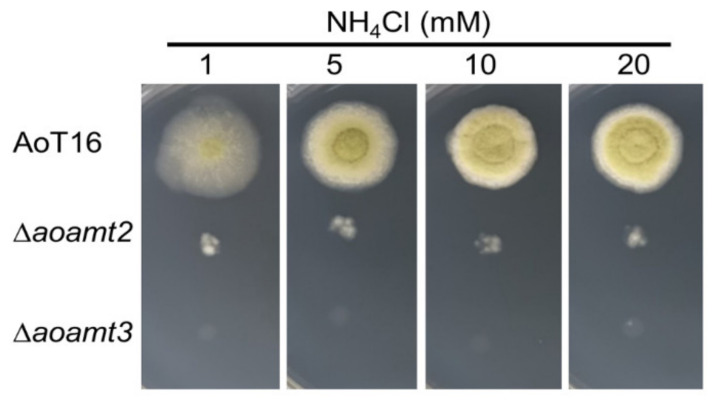
Radial growth analysis of the *aoamt*-disrupted strains of *A. oryzae*. Five µL aliquots of spore suspension (5000 spores) of the Δ*aoamt2* and Δ*aoamt3* strains were spotted on mCD medium (pH 4.5) containing 1, 5, 10, or 20 mM NH_4_Cl. The recipient (AoT16) was used as a control. Fungal cultures were incubated at 30 °C for three days.

To study the role of Aoamt protein in nitrogen metabolism, particularly in relation to ammonium transporting capacity and ammonium assimilation to glutamic acid/glutamine biosynthesis, genetic perturbation of *aoamt2* and *aoamt3* was performed. For gene disruption, the *aopyrG* auxotrophic marker was used for gene replacement with homologous recombination resulting in the prototrophic *aoamt* disruptants. The genetic modification of both transporter genes was verified by reverse transcription-polymerase chain reaction (RT-PCR) (Figure S2). The growth phenotypes of the disruptant strains cultivated on solid agar media containing various NH_4_Cl concentrations (1, 5, 10, and 20 mM) were assessed and compared to that of the recipient strain (prototroph). As seen in Figure 5, the *aoamt2* and *aoamt3* disruptant strains showed defective growth on mCD agar, and the increase in ammonium concentration did not support aerial growth development, in contrast to previous studies. The deletion of *amt3* and *mepB* (*aoamt3*-like) genes in *Schizosaccharomyces pombe* and *A. nidulans*, respectively, had no effect on cell growth at a wide range of ammonium concentrations tested [19,42]. Significantly, the loss of *aoamt3* gene function led to severe growth retardation, which is different from the observations in Δ*aoamt2* growth. The existing function of the Aoamt3 transporter may explain the survival of the *aoamt2* strain in ammonium. We suggest that the Aoamt3 transporter encoded by the *aoamt3* gene is responsible for the ammonium transport system in *A. oryzae*, which is significantly involved in the ammonium transport capacity required for cell growth. It seems that other ammonium transporters cannot compensate for the activity of Aoamt3 in the uptake of extracellular ammonium. These results also suggest that Aoamt2 might not mediate ammonium transport. Notably, only 1 mM NH_4_Cl was sufficient to differentiate the phenotypic growth of the AoT16 recipient, and Δ*aoamt2* and Δ*aoamt3* strains on a solid agar medium. Therefore, this concentration was used in subsequent experiments.

**Figure 6:**
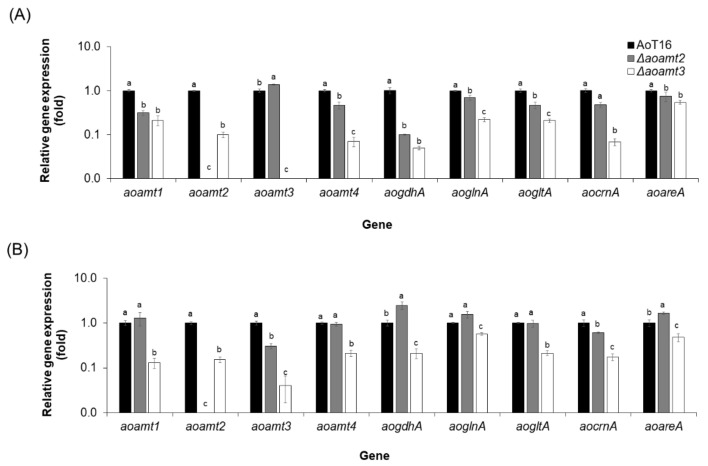
Transcriptional analysis of a set of genes in the *A. oryzae*-disrupted strains. Expression analysis of *aoamt1–4*, *aogdhA*, *aoglnA*, *aogltA*, *aocrnA*, and *aoareA* genes of the recipient (AoT16, black bar), Δ*aoamt2* (gray bar) and Δ*aoamt3* (white bar) strains was carried out by RT-qPCR. RNA samples were extracted from the cultures after transferring to a 1 mM NH_4_Cl-containing medium for 1 h (**A**) and 8 h (**B**), which were defined as ammonium deficient and starvation conditions, respectively. The gene expression level in the AoT16 was adjusted to 1 (black bars). Different letters (a, b and c) above the bars indicate a statistically significant difference in the transcript levels of each gene among the three strains analyzed by Duncan’s multiple range test (MRT) (*p* value < 0.05). The mean ± standard deviation (mean ± SD) of the expression level was derived from triplicate analysis.

**Figure 7:**
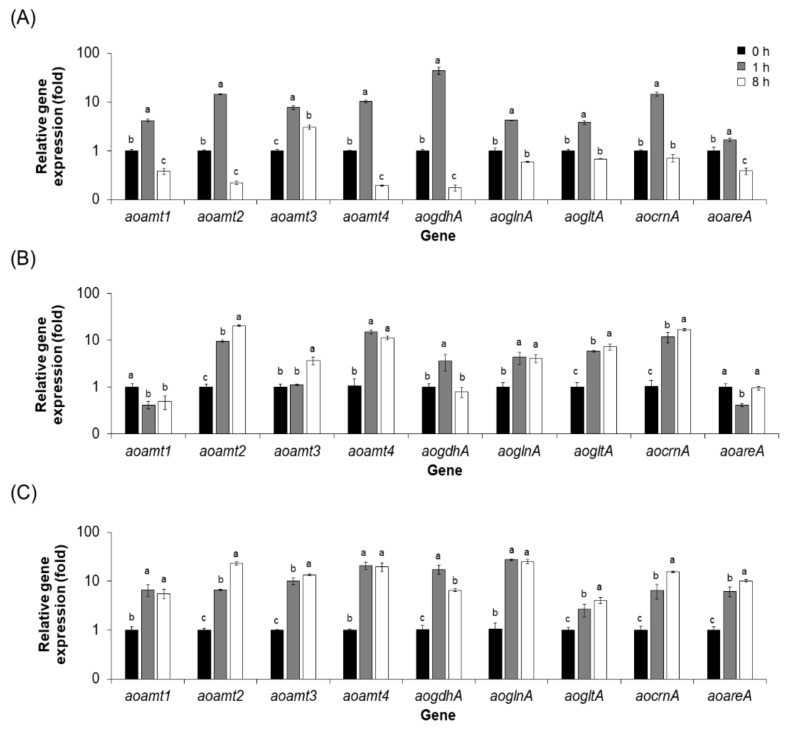
Transcriptional analysis of a set of genes in the overexpressed strains at different time points of cultivation. Expression analysis of *aoamt1–4*, *aogdhA*, *aoglnA*, *aogltA*, *aocrnA*, and *aoareA* genes in the AoT16 (**A**), *oeaoamt2* (**B**) and *oeaoamt3* (**C**) cultures grown in 1 mM NH_4_Cl-containing medium was carried out by RT-qPCR. RNA samples were extracted from the cultures after transferring to a 1 mM NH_4_Cl-containing medium for 0, 1, and 8 h. The expression level of each gene at 0 h (black bars) was adjusted to 1. Different letters (a, b, and c) above the bars indicate a statistically significant difference in the transcript levels of each gene at various time points analyzed by Duncan’s multiple range test (MRT) (*p* value < 0.05). The mean ± standard deviation (mean ± SD) of relative expression level is derived from triplicate analysis.

Using RT-qPCR, the transcriptional profiles of genes involved in the nitrogen metabolism, namely *aoamts*, *aogdhA*, *aoglnA*, *aogltA*, *aocrnA*, and *aoareaA*, of the disrupted and overexpressed strains were investigated after transferring the cultures to the medium broth containing 1 mM NH_4_Cl (Figure 6 and Figure 7). Ammonium deficient and starvation conditions for all tested strains were defined at 1 and 8 h of cultivation, respectively (Figure S1B–F). Compared with the recipient strain, upregulation of the *aoamt3* gene was found only in the Δ*aoamt2* strain under ammonium-deficient condition (gray bar in Figure 6A). Under starvation condition, the growth of the Δ*aoamt2* strain was also sustained because of the expression of genes involved in glutamic/glutamine biosynthesis and transcription factor AreA in regulation of nitrogen-associated metabolism (gray bars in Figure 6B). It was observed that the expression profiles of all tested genes were upregulated under deficient condition and downregulated equivalent to or lower than the expression level at 0 h (starvation condition) (Figure S4A). Defect in the *aoamt3* gene led to significant repression of all genes compared with that found in the AoT16 strain (white bars in Figure 6A,B and Figure S4B), particularly under ammonium starvation condition, leading to a lack of growth (Figure 5). This evidence confirms the importance of the *aoamt3* gene encoding Aoamt3 in ammonium uptake for cell growth and metabolism. The *mepB* (*aoamt3*-like) gene deletion in *S. pombe* showed no detectable effect on cell growth at all the ammonium concentrations tested [42]. The function of the *aoamt3* gene involved in ammonium transport was explored using gene disruption analysis. Gene complementation studies are also a potent approach for verifying gene function.

The overexpression of *aoamt2* and *aoamt3* was investigated at different time points during cultivation. RT-qPCR analysis showed constitutive expression of both genes in the *oeaoamt2* and *oeaoamt3* cultures as a result of the control of *toxA* promoter (Figure 7B,C). Interestingly, the expression of the *aoamt3* gene in the *oeaoamt3* strain was strongly upregulated, particularly under ammonium starvation condition (Figure 7C) compared with the AoT16 recipient (Figure 7A). It has been suggested that *aoamt3* is a key gene involved in nitrogen metabolism. It is likely that there was a cooperative regulation of several genes involved in ammonium transport at least at the transcription level to sustain cell growth and metabolism, that is *aoamt1–4*, *aoglnA*, *aogltA*, *aocrnA*, and *aoareA*. Reduced expression of the *aogdhA* gene was observed in the *oeaoamt3* strain grown under ammonium starvation condition, which may explain by the metabolic function of an alternative route of glutamic acid biosynthesis via the activity of AogltA [2].

Therefore, our results suggest the significance of the *aoamt3* gene, which is expressed in all stages of ammonium existing cultivation. The encoded protein transporter contributes to ammonium uptake. In contrast, the absence of the *aoamt3* gene resulted in severe growth defects. Taken together, the Aoamt3 transporter encoded by the *aoamt3* gene plays a significant role in ammonium transport and nitrogen metabolism in *A. oryzae*.

### 2.4. Effect of Aoamt3 Overexpression of on Cell Biomass Production of A. oryzae

**Figure 8:**
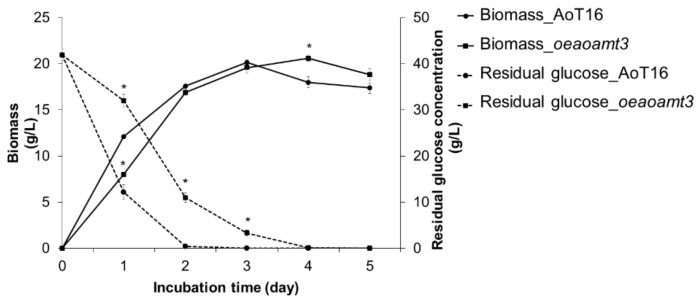
Comparative growth profiles of the *oeaoamt3* and AoT16 strains. Fungal cultures were grown in SM broth medium at 30 °C and 250 rpm, and the samples were taken at different cultivation times for analysis. (•) and (▪) represent the AoT16 and *oeaoamt3* strains, respectively. Biomass titer and residual glucose concentration are shown by solid and dashed lines, respectively. “*” above the symbols indicates a significant difference in residual glucose concentrations and biomass titers, respectively of the *oeaoamt3* and AoT16 strains (*p* < 0.05). Experiments were performed in triplicates.

From an industrial perspective, more speculation was then given to gene overexpression and the utilization of simple and inexpensive nitrogen sources to investigate the growth phenotypic alteration in terms of biomass production. The increase in the uptake capacity of basic and cheap nitrogen sources, such as ammonium and mixed nitrogen into cells with efficient glutamic acid biosynthesis, is a prospect for enhanced biomass production that would be beneficial in the development of the fungal production process, particularly in the production of growth-associated metabolites of a target. Using a mixture of nitrogen sources (NH_4_Cl and yeast extract) for submerged cultivation, the fungal growth and biomass titer of the *oeaoamt3* strain in the early phase were comparable to those of the wild type (Figure 8). However, biomass production was sustained and was higher than that of the wild type as clearly observed in the stationary phase. The maximum biomass titer was observed in the *oeaoamt3* culture grown for four days. Moreover, the period of complete glucose consumption of the *oeaoamt3* strain was prolonged compared with that of the wild type. This may be a result of constitutive overexpression of *aoamt3* leading to a continual increase in biomass until the starvation stage. The growth parameters of the *oeaoamt3* as well as the wild type strains were calculated from the cultures grown for two days, as shown in Table 1. Even though the biomass titer and productivity of both strains were not significantly different, the glucose consumption and glucose consumption rate of the overexpressing strain reduced by 26 and 24%, respectively, and the biomass yield increased by 24%. These results indicate that Aoamt3 is an attributive transporter for ammonium uptake, which is involved in cell growth and biomass production. Therefore, overexpression of *aoamt3* increases the ammonium transport capacity to sustain cell growth and biomass production with less glucose consumption.

## 3. Methods and Materials

### 3.1. Microbial Strains and Cultivation

The wild type *A. oryzae* strain BCC 7051 was used for expression analysis of the putative *aoamt* genes identified. A *pyrG*-deficient strain (auxotroph) of *A. oryzae* was used to generate recombinant strains by gene disruption and overexpression. The *pyrG* gene complementation strain (prototroph) was used as a recipient for comparison with the recombinant strains in terms of growth phenotypes on solid cultivation and gene expression. Czapek Dox (CD) medium (BD Difco) was used to maintain the fungal cultures. The deficient strain was cultivated by supplementation with 5.0 g/L uridine and 0.2 g/L uracil. For submerged cultivation, spore inoculum was prepared by cultivating mycelial cells on sterile rice for 5 days. Spores were harvested by suspension with 0.05% (*v*/*v*) Tween 80 solution, filtered using Miracloth (MerckMillipore, Darmstadt, Germany), and centrifuged. The spore suspension (10^8^ spores) was inoculated into 50 mL of appropriate culture medium.

*Escherichia coli* DH5α (Thermo Fisher Scientific, Waltham, MA, USA) (*supE44*, *DlacU169*, (*F80*, *lacZDM15*), *hsdR17*, *recA1*, *endA1*, *gyrA96*, *thi1*, *relA1*) was used for plasmid propagation. Bacterial cells were grown in Luria-Bertani (LB) medium (BD Difco) containing 100 mg/L ampicillin at 37 °C with shaking at 200 rpm.

*Saccharomyces cerevisiae* strain INVSCI (Invitrogen, Waltham, MA, USA) (*MAT*α, *his3-D1*, *leu2*, *trp1-289*, *ura3-52*, *MAT*, *his3-D1*, *leu2*, *trp1-289*, *ura3-52*) was used for DNA assembly. Yeast culture was routinely grown on yeast extract peptone dextrose (YPD) medium containing 1% bacto-yeast extract, 2% bacto peptone, and 2% glucose at 30 °C. For transformant selection, yeast cells were grown on the Synthetic Defined (SD) agar medium (BD Difco) consisting of 0.67% (*w*/*v*) yeast nitrogen base without amino acids, 2% (*w*/*v*) glucose, and 2% (*w*/*v*) agar supplemented with L-tryptophan, L-histidine-HCl, and L-leucine at concentrations of 20, 20 and 30 mg/l, respectively.

### 3.2. Identification of Aoamt Homologs of A. oryzae

To obtain the putative genes encoding ammonium transporters of *A. oryzae* (*aoamts*), a BLAST search against the genome sequence of strain BCC 7051 [23] was performed using the NCBI database (http://www.ncbi.nlm.nih.gov/, accessed on 30 November 2021) with the *amt* sequences of *Aspergillus flavus* as queries [43]. The cDNA cloning of these putative genes was carried out by RT-PCR using 50 ng of total RNA from *A. oryzae* BCC 7051 as a template, specific primer sets (Table S1), and the Super Script III One-Step RT-PCR with Platinum *Taq* DNA Polymerase (Invitrogen, Waltham, CA, USA). The amplified cDNA fragments were purified using the QIAquick Gel Extraction kit (Qiagen, Germany) and subcloned into the TOPO 2.1 vector (TOPO^®^ TA cloning^®^ kit, Invitrogen). Sequence analysis was performed using by Macrogen (Seoul, South Korea). The four cDNA sequences obtained, *aoamt1*, *aoamt2*, *aoamt3*, and *aoamt4*, were deposited in the GenBank database (http://www.ncbi.nlm.nih.gov, accessed on 30 March 2022) under the accession numbers ON098152, ON098153, ON098154, and ON098155, respectively.

### 3.3. Transmembrane Topology Prediction

To analyze the transmembrane protein topology of the individual *A. oryzae* BCC 7051 ammonium transporters, the protein sequences were predicted using TMHMM 2.0 [24] based on a hidden Markov model. For predicting results, the program presents the number and location of predicted transmembrane helix features involved in the charge, polarity, and hydrophobicity of each protein sequence.

### 3.4. Analysis of Phylogenetic Tree and Conserved Motifs of the Aoamt Proteins

Full-length amino acid sequences of the ammonium transporters of *A. oryzae* BCC 7051, yeast, and other filamentous fungi were retrieved from the NCBI database using BLASTP. Multiple sequence alignment of the amino acid sequences was performed to calculate the genetic distance using the Clustal algorithm with the default parameter settings [44]. For phylogenetic analysis, the alignment results were imported into the Molecular Evolutionary Genetics Analysis software (MEGA, version 11) [45]. The optimal model of phylogenetic relationship was determined using the Find Best Protein Model’ option provided by MEGA. Subsequently, the evolutionary tree was generated using the maximum-likelihood method with a different amino acid frequency (+F) model [46], in which a discrete gamma distribution (+G) with five rate categories is under the assumption that a certain fraction of sites is evolutionarily invariable (+I). These sites were selected as the best-fit substitution model for the AMT/MEP family sequence evolution. Additionally, all positions containing gaps and missing data were eliminated from the dataset (complete deletion). The confidence intervals of the phylogenetic trees were tested using the bootstrap statistical method [47] with 500 resampling iterations, in which the initial trees were automatically obtained.

The conserved motifs among the AMP/MEP proteins of *Aspergilli* were identified by comparing amino acid sequences between these proteins and protein family databases using Pfam release 35.0 at the Sanger Centre, UK (http://pfam.xfam.org/, accessed on 4 February 2022) [48]. The functional regions of the query proteins were searched against the domains Pfam-A and Pfam-B using a default E-value cut-off of 1.0, which was defined based on expert knowledge, sequence similarity, other protein family databases, and the ability of HMM profiles to correctly identify and align the members. Conservative sequence motifs were extracted from the amino acid sequence alignment using the multiple sequence alignment program from the Clustal Omega package (http://www.clustal.org/omega/, accessed on 14 February 2022) [49].

### 3.5. Plasmid Construction, Fungal Transformation, and Verification of Transformants

A scheme summarizing the plasmid construction is presented in Figure S3. All plasmids were constructed using PCR and DNA assembly in yeast cells [50]. To construct the disruption plasmid, pDaoamt2 or pDaoamt3 (Figure S3A), the specific 5′ and 3′ DNA fragments with homologous sequences of individual *aoamt* genes were amplified using the genomic DNA template of *A. oryzae* BCC 7051, Platinum^TM^ Tag DNA polymerase Hi-Fidelity (Invitrogen) and overlapping primer sets (Table S2). DNA assembly of the homologous fragments and linearized backbone plasmid BPO152-carrying *aopyrG* marker was performed in *S. cerevisiae*. Plasmids were extracted from the yeast transformants using the Zymoprep^TM^ Yeast plasmid Miniprep I (Zymo Research, Irvine, CA, USA) and were then shuttled into *E. coli* cells. The constructed plasmids were confirmed using DNA sequencing. To generate the overexpression plasmids, poeaoamt2 and poeaomt3 (Figure S3B), the constitutive *toxA* promoter and *nos3* terminator fragments were amplified from the plasmid pCT74 [51], and the targeted *aoamt* cDNAs were derived from the cloning plasmids using overlapping primers (Table S2). Then, DNA assembly of these fragments with the linearized backbone plasmid BPO152 was performed in yeast cells. The plasmid or DNA fragment (2–5 μg) was transformed into protoplast cells using the PEG-mediated method (PMT) [52]. Transformants were selected on CD medium after incubation at 30 °C for 3–7 d. Spore re-isolation was performed to obtain a pure culture. To verify gene disruption or overexpression, total RNA extracted from the fungal cultures was subjected to the Super Script III One-Step RT-PCR with Platinum *Taq* DNA Polymerase (Invitrogen, Waltham, CA, USA) using specific oligonucleotide primers (Table S3).

### 3.6. Submerged Cultivation for Gene Expression Analysis and Determination of Ammonium Consumption

To study differential gene expression and ammonium consumption, fungal cultivation was performed with some modifications from the published method [18], in which the logarithmic culture was transferred to ammonium-lacking medium, followed by ammonium-containing medium at different concentrations. The spore suspension (10^8^ spores) was inoculated into a modified CD (mCD) medium (10 g/L (*w*/*v*) glucose, 5 g/L (*w*/*v*) yeast extract, 1 g/L (*w*/*v*) K_2_HPO_4_, 0.5 g/L (*w*/*v*) MgSO_4_.7H_2_O, 0.5 g/L (*w*/*v*) KCl, 15 mg/L (*w*/*v*) FeCl_3_·7H_2_O, 10 mg/L (*w*/*v*) MnSO_4_·H_2_O, and 7.5 mg/L (*w*/*v*) ZnSO_4_·7H_2_O) with initial pH 4.5 as a basal medium. The fungal culture was carried out at 30 °C with shaking for 16 h. Mycelial cells were harvested, washed twice with sterile distilled water, and resuspended in mCD medium without nitrogen (nitrogen-free medium) at 30 °C with shaking for 4 h. Then, the fungal mycelia were transferred to ammonium-containing medium by changing the nitrogen source in the basal mCD medium, in which the yeast extract was replaced with 1 or 20 mM NH_4_Cl (Sigma-Aldrich, St. Louis, MO, USA) as a sole nitrogen source and incubated at 30 °C with shaking. The culture samples were harvested at different time points.

### 3.7. RT-qPCR Analysis

Total RNA was extracted from harvested mycelial cells using a PureLinkTM RNA Mini kit (Invitrogen). Reverse transcription of total RNA to cDNA was performed using the RevertAid First Strand cDNA Synthesis Kit (Thermo Fisher Scientific). RT-qPCR was conducted according to a previously described method [53] using the Luna^®^ Universal qPCR Master Mix (New England BioLabs, Hitchin, UK) and specific primer sets (Table S3). Relative quantification of gene expression was performed by normalization to 18S rRNA [54]. Individual experiments were independently performed in triplicates.

### 3.8. Radial Growth and Cell Biomass Measurements

To investigate mycelial growth on solid agar, a spore inoculum (5000 spores) of the recipient or disruptant strains was dropped onto basal agar media containing 1, 5, 10, and 20 mM NH_4_Cl and incubated at 30 °C for 3 days. Each experiment was performed with three technical replicates.

To determine biomass production of the *oeaoamt3* strain, cultivation was performed by inoculating spores into a semi-synthetic medium, SM (40 g/L (*w*/*v*) glucose, 0.2 g/L (*w*/*v*) NH_4_Cl, 5 g/L (*w*/*v*) yeast extract, 2.4 g/L (*w*/*v*) KH_2_PO_4_, 0.5 g/L (*w*/*v*) MgSO_4_·7H_2_O, 0.1 g/L (*w*/*v*) CaCl·2H_2_O, 15 mg/L (*w*/*v*) FeCl_3_·7H_2_O, 10 mg/L (*w*/*v*) MnSO_4_·H_2_O, and 7.5 mg/L (*w*/*v*) ZnSO_4_·7H_2_O) [55]. Mycelial samples were harvested at different time points during cultivation, collected by filtration with gentle suction, washed with sterile water, and dried in a hot-air oven until a constant weight was obtained. The biomass titer was represented as the dry cell weight per liter (DCW/L).

### 3.9. Determination of Residual Glucose and Ammonium Concentrations

The residual glucose concentration in the fermented broth was measured using high-performance liquid chromatography (HPLC). The analysis was performed at 60 °C with a flow rate of 0.6 mL/min on an Aminex HPX-87H column (9 µm particle size, 300 × 7.8 mm, Bio-Rad, Hercules, CA, USA), and 18 mM sulfuric acid was used as the mobile phase. A calibration curve of glucose concentration was generated to calculate the residual glucose concentration in the fermented broth samples.

Ammonium concentration was determined calorimetrically using the phenol-hypochlorite method with some modifications from a published method [56]. The filtration broth (0.1 mL) of the culture was thoroughly mixed with 0.5 mL of phenol (5%, *v*/*v*) and nitroprusside (0.005%, *w*/*v*), and then 0.5 mL alkaline hypochlorite solution (0.5% (*w*/*v*) sodium hydroxide, and 0.8% (*w*/*v*) sodium hypochlorite) was added. The mixture was incubated at 37 °C for 20 min and subjected to measure the colorimetric absorbance was measured at 625 nm. A calibration curve was obtained by using the ammonium chloride at various concentrations (0.1–0.5 mM was the standard). The ammonium concentration in the culture broth sample was reported as millimolar (mM).

### 3.10. Statistical Analyses

Statistical analysis was performed using SPSS version 11.5 software for Windows (SPSS Inc., Chacago, IL, USA). Three replicates were established for each experiment. The results are presented as mean values with standard deviation (mean ± SD). The data were subjected to one-way analysis of variance (ANOVA) and compared using Duncan’s multiple range test (DMRT) with a statistically significant difference (*p*-value < 0.05) for multiple data sets or independent sample *t*-test at a 95% confidence interval for two sample means.

## 4. Conclusions

Four ammonium transporters of *A. oryzae* (*aoamt1–4*), belonging to the AMT/MEP family, were identified and characterized. Differential expression analysis of these genes indicated that the expression of *aoamt2*, *aoamt3*, and *aoamt4* was ammonium-dependent. Aoamt2 was highly upregulated in ammonium-deficient, whereas the upregulation of *aoamt3* was prolonged under starvation conditions. The role of these transporters in nitrogen metabolism was explored by gene disruption and overexpression, which showed that Aoamt3 is the major ammonium transporter required for cell growth and has an impact on the nitrogen metabolism of *A. oryzae*. Moreover, overexpression of *aoamt3* enhanced the overall biomass production with a lower glucose consumption rate than that of the wild type. These results provide insight into the molecular mechanisms of nitrogen metabolism underlying the ammonium transport capacity of Aoamt3 in *A. oryzae*, which offers rational improvement in cell biomass production by either physiological control or synthetic biology approaches.

## Figures and Tables

**Table 1:** Comparison of growth parameters between the overexpressed strain (*oeaoamt3*) and the AoT16 recipient of *A. oryzae*.

Growth Parameter	*oeaoamt3*	AoT16
Biomass (g/L)	16.91 ± 0.34	17.60 ± 0.13
Productivity (g/L/h)	0.35 ± 0.01	0.37 ± 0.00
Glucose consumption (g/L)	30.85 ± 1.08 *	41.46 ± 0.18
Glucose consumption rate (g/h)	0.65 ± 0.01 *	0.86 ± 0.00
Biomass yield (g cell/g substrate)	0.55 ± 0.01 *	0.42 ± 0.00

“*” indicates a significant difference in values of *oeaoamt3* and the AoT16 strains by independent sample *t*-test at a 95% confident interval. The experiments were carried out in triplicates.

## Data Availability

The data presented in this study are available on request from the corresponding author.

## Supplementary Materials

The following supporting information can be downloaded at: https://www.mdpi.com/article/10.3390/ijms23147567/s1.
