# Supplementary material for: The Exploring Functional Role of Ammonium Transporters of Aspergillus oryzae in Nitrogen Metabolism: Challenges towards Cell Biomass Production
Source: Int J Mol Sci. 2022 Jul 8;23(14):7567. doi: 10.3390/ijms23147567 (PMC9319855; doi:10.3390/ijms23147567)
Supplement: Supplementary file 1 [file ijms-23-07567-s001.zip › ijms-1762738-supplementary.pdf]

Supplementary materials

The Exploring Functional Role of Ammonium Transporters of *Aspergillus oryzae* in Nitrogen Metabolism: Challenges towards Cell Biomass Production

Chanikul Chutrakul [1,*], Sarocha Panchanawaporn [1], Tayvich Vorapreeda [2], Sukanya Jeennor [1], Jutamas Anantayanon [1] and Kobkul Laoteng [1]

[1] Functional Ingredients and Food Innovation Research Group (IFIG), National Center for Genetic Engineering and Biotechnology (BIOTEC), National Science and Technology Development Agency (NSTDA), Thailand Science Park, Phahonyothin Road, Khlong Nueng, Khlong Luang, Pathum Thani 12120, Thailand; chanikul@biotec.or.th (C.C.), sarocha.pan@biotec.or.th (S.P.), sukanya.jee@biotec.or.th (S.J.), jutamas.ana@biotec.or.th (J.A.), kobkul@biotec.or.th (K.L.)

[2] Biochemical Engineering and Systems Biology Research Group (IBEG), National Center for Genetic Engineering and Biotechnology (BIOTEC), National Science and Technology Development Agency (NSTDA), at King Mongkut's University of Technology Thonburi, Bangkok 10150, Thailand; tayvich.vor@biotec.or.th

* Correspondence: chanikul@biotec.or.th; Tel.: +66-25646700 Ext. 3769

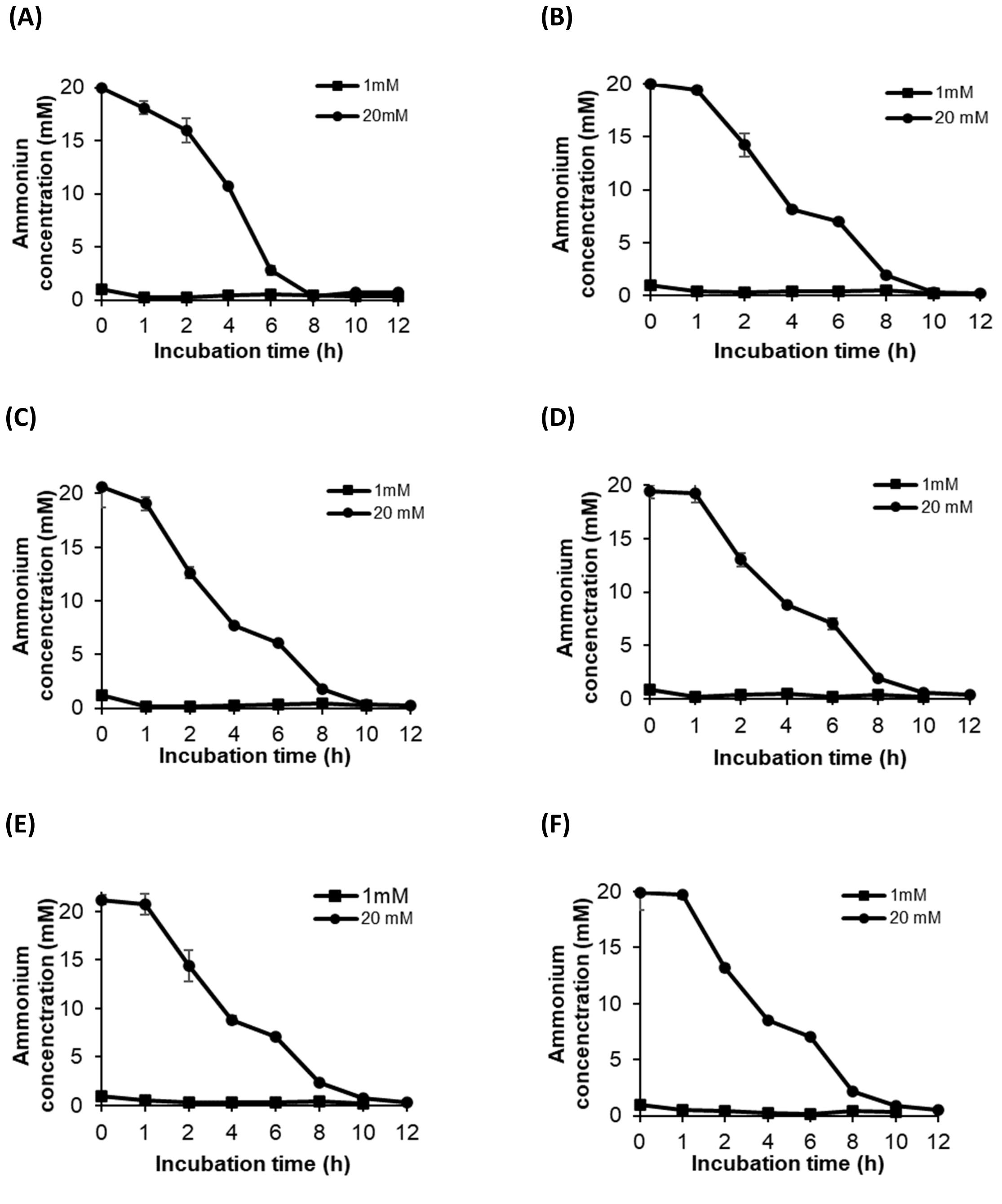

Figure S1. Residual ammonium concentration in the *A. oryzae* cultures. The spore suspension was inoculated in modified Czapek Dox (mCD) broth medium for 16 h, and prolonged cultivation for 4 h in nitrogen-free mCD was carried out before transferring the cultures into 1 or 20 mM NH_4Cl-containing medium. Culture samples of wild-type (**A**), AoT16 (**B**), *Δaoamt2* (**C**), *oeaoamt2* (**D**), *Δaoamt3* (E), and *oeaoamt3* (F) were collected at different time points to measure the ammonium concentration. Experiments were carried out in triplicates.

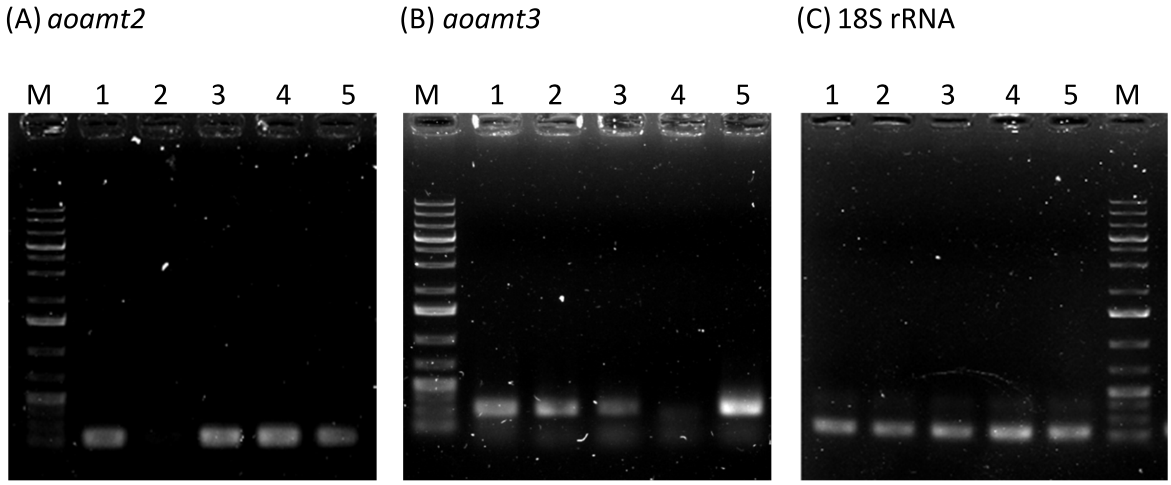

Figure S2. Verification of deletion of *aoamt2* and *aoamt3* in *A. oryzae* disruptant strains. Detection of *aoamt2* (**A**) and *aoamt3* (**B**) genes was performed by RT-PCR. Total RNA templates were prepared from the cultures of the recipient (AoT16) (lane 1), *Δaoamt2* (lane 2), *oeaoamt2* (lane 3), *Δaoamt3* (lane 4), and *oeaoamt3* (lane 5) strains grown in SM medium for 24 h. Lane M is 1 kb DNA plus ladder (Thermo Fisher Scientific).

(A)

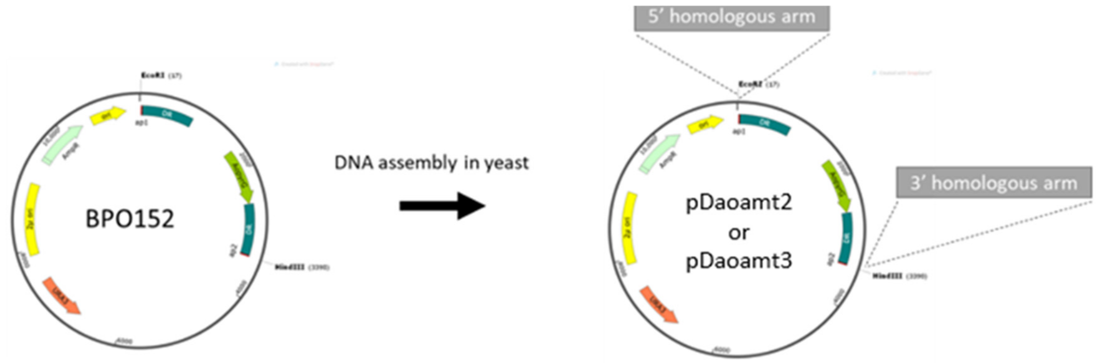

(B)

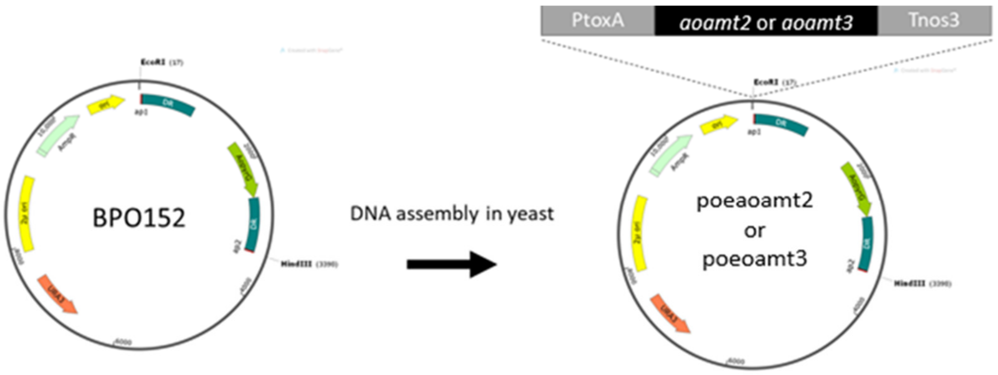

Figure S3. Schematic construction of plasmids for disruption and overexpression of *aoamt* gene in *A. oryzae*. (**A**) The pDaoamt2 or pDaoamt2 disrupted plasmid containing the *aopyrG* marker cassette with 5' and 3' homologous fragments for targeted recombination with individual *aoamt* genes. (**B**) The poeaoamt2 or poeaoamt3 overexpression plasmids containing the expression cassette of individual *aoamt* genes.

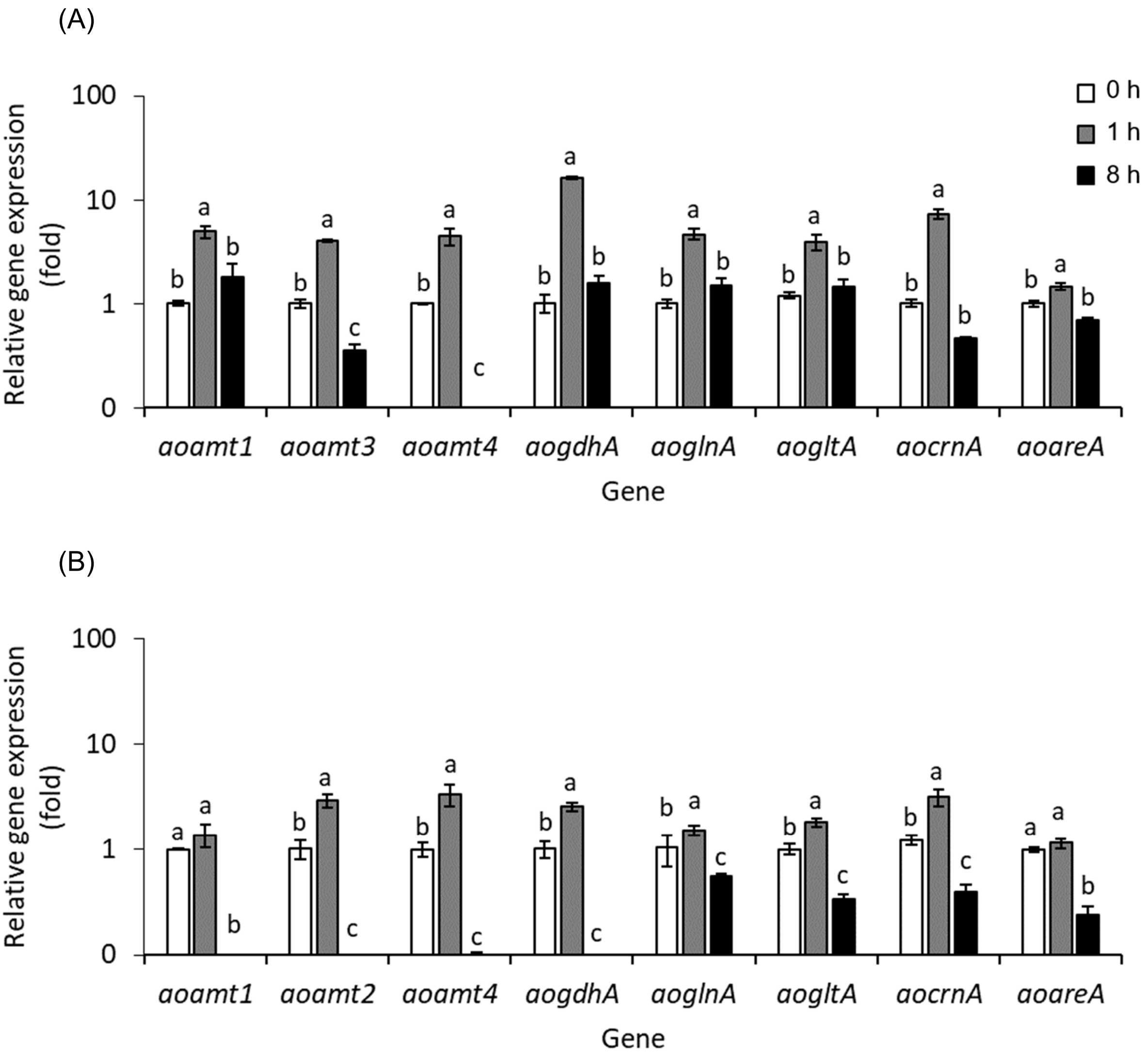

Figure S4. Expression analysis of a set of genes in disrupted strains at different cultivation times by RT-qPCR. Relative expression levels of *aoamt1-4*, *aogdhA*, *aoglnA*, *aogltA*, *aocrnA*, and *aoareA* of *Δaoamt2* (**A**) and *Δaoamt3* (**B**) strains grown in 1 mM NH_4Cl-containing medium are illustrated. Total RNA was extracted from the cultures after transferring them to a 1 mM NH_4Cl-containing medium for 0, 1, and 8 h. The expression level of each gene at 0 h (white bars) is adjusted to 1. Different letters (a, b, and c) above the bars indicate a statistically significant difference in the transcript levels of each gene at various time points, analyzed by Duncan's multiple range test (MRT) (p-value < 0.05). The mean ± standard deviation (mean ± SD) of the relative expression level analyzed in triplicates is presented.

Table S1. Specific oligonucleotide primers used for cDNA cloning of ammonium transporter genes of *A. oryzae*

Gene	**Sense primer name**	**Sequence (5' to 3')**	**Antisense primer name**	**Sequence (5' to 3')**
aoamt1	Aoamt1_F	ATGTCGGACATCAAGGCGCCCTTC	Aoamt1_R	TTACTGTGTTTTCTCATCCTCCTCGACAC
aoamt2	Aoamt2_F	ATGGCAGAATACCCTGTGGCCTAC	Aoamt2_R	CTAAGCCTTGGCCTCTATGGTCGTTTC
aoamt3	Aoamt3_F	ATGGTCGCGCCGGTGTACAATGC	Aoamt3_R	CTAGACTCCTGGTGTTTTCACTGTTTGC
aoamt4	Aoamt4_F	ATGTCTGTCCAGGCTGCCTGGGAG	Aoamt4_R	CTAAAGCCGAACACCCTCAAAAGGATG

Table S2. Overlapping primers used for recombinant plasmid construction by PCR

Plasmid	Amplified fragment	Sense primer name	Sequence (5' to 3')	Antisense primer name	Sequence (5' to 3')
pDaoamt2	5'HR_ aoamt2	5'HR-Aoamt2-F	GGCCGATTCATTCCCGGAAGGCGCGCCGTCAATGGAGAGTGATTGATCAG	5'HR-Aoamt2-R	GGTACCTAGCTAGTTAGCAAGAATTCACTGTGGCTGTCGTTGAACAACG
	3'HR_ aoamt2	3'HR-Aoamt2-F	AAGTACCTACGTACGTACGGACTTAAGCTTCAGGAAGCGTCTAGTTGAAGACC	3'HR-Aoamt2-R	TTGTAAAACGGCGGGATCGCGGCGCGCCTAGGTAATCGTAGGATGTCGC
pDaoamt3	5'HR_aoamt3	5'HR-Aoamt3-F	GGCCGATTCATTCCCGGCGATCGCCTGTCTTGCCATTCTTGGTGAC	5'HR-Aoamt3-R	CGAATTCGTTTTGCTGGCCGCATCTGACCATGGCGAGCAGGCTCACTCTG
	3'HR_ aoamt3	3'HR-Aoamt3-F	AAGTACCTACGTACGTACGGACTTAAGCTTGTCTAGCGGTCGTGTTCGAATTAGG	3'HR-Aoamt3-R	GATCCCCGGGTACCGAGCTCGCGATCGCCAATCAGGCAACAAGAAGTCGG
poeaoamt2	PtoxA	PtoxA-Aoamt2-F	ACCCTACGTATCCAGATGAGCGGGTGGTATCGATTGGAATGCATGGAGGA	PtoxA-Aoamt2-R	TGTAGGCCACAGGGTATTCTGCCATGACCTATATTCATTCATTGTCAGCT
	aoamt2	Aoamt2-F	AGCTGACAATGAATGAATATAGGTCATGGCAGAATACCCTGTGGCCTACA	Aoamt2-R	GTTTGAACGATCTGCAGCCGGGCGGCTAAGCCTTGGCCTCTATGGTCGTT
	Tnos3	Tnos3-Aoamt2-F	AACGACCATAGAGGCCAAGGCTTAGCCGCCCGGCTGCAGATCGTTCAAAC	Tnos3-Aoamt2-R	GTAAAACGACGGCGGGATCGCAAGCTTAATTAATTCTCATGTTTGACAGCTTATCA
poeaoamt3	PtoxA	PtoxA-Aoamt3-F	ACCCTACGTATCCAGATGAGCGGGTGGTATCGATTGGAATGCATGGAGGA	PtoxA-Aoamt3-R	GAAGCATTGTACACCGGCGCGACCATGACCTATATTCATTCATTGTCAGCT
	aoamt3	Aoamt3-F	AGCTGACAATGAATGAATATAGGTCATGGTCGCGCCGGTGTACAATGCTTC	Aoamt3-R	GTTTGAACGATCTGCAGCCGGGCGGCTAGACTCCTGGTGTTTTCACTGTT
	Tnos3	Tnos3-Aoamt3-F	AACAGTGAAAACACCAGGAGTCTAGCCGCCCGGCTGCAGATCGTTCAAAC	Tnos3-Aoamt3-R	GTAAAACGACGGCGGGATCGCAAGCTTAATTAATTCTCATGTTTGACAGCTTATCA

Table S3. Specific oligonucleotide primers used for RT-qPCR

Gene	Sense primer name	Sequence (5' to 3')	Antisense primer name	Sequence (5' to 3')
aoamt1	Aoamt1_RT_F	TATGCGTCTTACAACTGGACTGGAGC	Aoamt1_RT_R	TACCATTGGAACGTGACGACAGAG
aoamt2	Aoamt2_RT_F	TCCTGGGTGCGCCATCTGTTGGCA	Aoamt2_RT_R	GCTCGAGTTCCAGGTCCAGCATGC
aoamt3	Aoamt3_RT_F	GGATCGCCTTCCTCTACAG	Aoamt3_RT_R	GCTGGAATAGGCAGAAGACG
aoamt4	Aoamt4_RT_F	CATATTGGCGTCCTTGCTGAACCG	Aoamt4_RT_R	AAGCCATTCTCTGACCAGACCATGTG
aogdhA	AogdhA_RT_F	ATGTCCAACCTTCCCATTGAGC	AogdhA_RT_R	AGCGGAGTTGAACTGAACACG
aoglnA	AoglnA_RT_F	CTACCGTGCTTGCTTGTACGC	AoglnA_RT_R	GGCGACGGTGGAGACGTTGC
aogltA	AogltA_RT_F	CAAGGATATGGCTGGGCTAATAC	AogltA_RT_R	CGTCATCACACCGGCACCATC
aocrnA	AocrnA_RT_F	GTCCCTTGCCAAGTCTGGTGTAC	AocrnA_RT_R	TCCTGTTGGGGTGTCTTCGC
aoareA	AoareA_RT_F	TGCCCAACCAGGAGCGTATGG	AoareA_RT_R	AATCTTGACGGCACTGGGCGAG
18S rRNA	18S rRNA_RT_F	GTAACCCGTTGAACCCCATT	18S rRNA_RT_R	CCATCCAATCGGTAGTAGCG
